# Socioeconomic Status and Overall Satisfaction with the Healthcare Among Older Adults in the U.S.

**DOI:** 10.1093/geroni/igaf122.390

**Published:** 2025-12-31

**Authors:** Rui Huang, Yaqi Yuan, Yulin Yang

**Affiliations:** University at Buffalo, Buffalo, New York, United States; Georgia Institute of Technology, Atlanta, Georgia, United States; University of California, San Francisco, San Francisco, California, United States

## Abstract

This study investigates the relationship between socioeconomic status (SES) and healthcare satisfaction among older adults, with a particular emphasis on how age moderates this relationship. Although substantial research has paid attention to health inequalities, there remains limited evidence on how SES factors such as education and wealth affect healthcare satisfaction longitudinally as individuals grow older. Guided by the Fundamental Cause Theory and Cumulative Inequality theory, this study explores whether and how education and wealth predict disparities in healthcare satisfaction over time and examines the moderating effect of age on these relationships. Data from four waves (2014, 2016, 2018, and 2020) of the Health and Retirement Study (HRS) were analyzed using multilevel logistic models with random intercepts and effects to capture within- and between-individual variabilities. The key independent variables include total wealth and educational attainment. Self-reported healthcare satisfaction is the dependent variable. Age is the moderator. Control variables include demographic characteristics, Medicare coverage, and self-rated health status. Preliminary results indicate that the association between SES and healthcare satisfaction is significantly influenced by age. Specifically, older adults with higher levels of education and greater wealth exhibit increasing satisfaction with healthcare as they age compared to their lower-income and less-educated peers, highlighting a widening gap in healthcare experiences across socioeconomic lines. Policymakers should prioritize targeted interventions to reduce healthcare disparities among aging populations by addressing socioeconomic barriers. Expanding equitable access to high-quality healthcare services, improving health literacy, and increasing financial protection for lower-income older adults may mitigate the adverse effects of socioeconomic disadvantages.

